# The Montevideo Interpretation of Quantum Mechanics: A Short Review

**DOI:** 10.3390/e20060413

**Published:** 2018-05-29

**Authors:** Rodolfo Gambini, Jorge Pullin

**Affiliations:** 1 Instituto de Física, Facultad de Ciencias, Iguá 4225, esq. Mataojo, Montevideo 11400, Uruguay; 2 Department of Physics and Astronomy, Louisiana State University, Baton Rouge, LA 70803-4001, USA

**Keywords:** quantum mechanics, decoherence, interpretations

## Abstract

The Montevideo interpretation of quantum mechanics, which consists of supplementing environmental decoherence with fundamental limitations in measurement stemming from gravity, has been described in several publications. However, some of them appeared before the full picture provided by the interpretation was developed. As such, it can be difficult to get a good understanding via the published literature. Here, we summarize it in a self-contained brief presentation including all its principal elements.

## 1. Introduction: The Measurement Problem

Although quantum mechanics is a well-defined theory in terms of providing unambiguous experimental predictions that can be tested, several physicists and philosophers of science find its presentation to be unsatisfactory. At the center of the controversy is the well-known measurement problem. In the quantum theory, states evolve unitarily, unless a measurement takes place. During a measurement, the state suffers a reduction that is not described by a unitary operator. In traditional formulations, this non-unitary evolution is postulated. Such an approach makes the theory complete from a calculational point of view. However, one is left with an odd formulation: a theory that claims our world is quantum in nature, yet its own definition requires referring to a classical world, as measurements are supposed to take place when the system under study interacts with a classical measurement device.

More recently, a more careful inspection of how the interaction with a measurement device takes place has led to a potential solution to the problem. In the decoherence program (for a review and references, see [1,2]), the interaction with a measurement device and, more generally, an environment with a large number of degrees of freedom, leads the quantum system to behave almost as if a reduction had taken place. Essentially, the large number of degrees of freedom of the measurement device and environment “smother” the quantum behavior of the system under study. The evolution of the combined system plus measurement device plus environment is unitary, and everything is ruled by quantum mechanics. However, if one concentrates on the wavefunction of the system under study only, tracing out the environmental degrees of freedom, the evolution appears to be non-unitary and very close to a reduction.

The decoherence program, suitably supplemented by an ontology like the many worlds one, has not convinced everyone (see for instance [3,4]) that it provides a complete solution to the measurement problem. Objections can be summarized in two main points:

1. Since the evolution of the system plus environment plus measuring device is unitary, it could happen that the quantum coherence of the system being studied could be recovered. Model calculations show that such “revivals” could happen, but they would take a long time for most realistic measuring devices. However, it is therefore clear that the picture that emerges is slightly different from the traditional formulation where one can never dial back a reduction. A possible answer is that for most real experimental situations, one would have to wait longer than the age of the universe. Related to this is the point of when exactly does the measurement take place? Since all quantum states throughout the evolution are unitarily equivalent, what distinguishes the moment when the measurement takes place? Some have put this as: “in this picture nothing ever happens”. A possible response is that after a certain amount of time, the state of the system is indistinguishable from the result of a reduction “for all practical purposes” (FAPP) [5]. However, from a conceptual point of view, the formulation of a theory should not rely on practical aspects. One could imagine that future scientists could perhaps find more accurate ways of measuring things and be able to distinguish what today is “FAPP” indistinguishable from a reduction.
  A related point is that one can define global observables for the system plus measuring device plus environment [3,6]. The expectation value for one of these observables takes different values if a collapse takes place or not. That could allow in principle to distinguish the FAPP picture of decoherence from a real collapse. From the FAPP perspective, the answer is that these types of observables are very difficult to measure, since this requires measuring the many degrees of freedom of the environment. However, the mere possibility of measuring these observables is not consistent with a realistic description. This point has recently been highlighted by Frauchiger and Renner [7], who show that quantum mechanics is inconsistent with single world interpretations.
2. The “and/or” problem [8]: Even though the interaction with the environment creates a reduced density matrix for the system that has an approximate diagonal form, as all quantum states, the density matrix still represents a superposition of coexisting alternatives. Why is one to interpret it as exclusive alternatives with given probabilities? When is one to transition from an improper to a proper mixture, in d’Espagnat’s terminology [3].
  The Montevideo interpretation [9] seeks to address these two criticisms. In the spirit of the decoherence program, it examines more finely what is happening in a measurement and how the theory is being formulated. It also brings into play the role of gravity in physics. It may be surprising that gravity has something to do with the formulation of quantum mechanics as one can imagine many systems where quantum effects are very important, but gravity seems to play no role. However, if one believes in the unity of physics, it should not be surprising that at some level, one needs to include all of physics to make certain situations work. More importantly, gravity brings to bear on physics important limitations on what can be done. Non-gravitational physics allows one to consider in principle arbitrarily large amounts of energy in a confined region, which is clearly not feasible physically if one includes gravity. This in particular places limitations on the accuracy with which we can measure any physical quantity [10,11]. Gravity also imposes limitations on our notions of space and time, which are absolute in non-gravitational physics. In particular, one has to construct measurements of space and time using real physical (and in this context, really quantum) objects, as no externally-defined space-time is pre-existent. This forces subtle changes in how theories are formulated. In particular, unitary theories do not appear to behave entirely unitarily since the notion of unitary evolution is defined with respect to a perfect classical time that cannot be approximated with arbitrary accuracy by a real (quantum) clock [12,13]. Notice that the role of gravity in this approach is different than in Penrose’s [14]. Here, the emphasis is on limitations to clocks due to the intrinsically relational nature of time in gravity, whereas in Penrose’s differences in time in different places is what is the basis of the mechanism.

These two new elements that the consideration of gravity brings to bear on physics will be key in addressing the two objections to decoherence that we outlined above. Since the evolution of systems is not perfectly unitary, it will not help to revive coherence in quantum systems to wait. Far from seeing coherence restored, it will be progressively further lost. The limitations on measurement will impose fundamental constraints on future physicists in developing means of distinguishing the quantum states produced by decoherence from those produced by a reduction. It will also make it impossible to measure global observables that may tell us if a reduction took place or not. Notice that this is not FAPP: the limitations are fundamental. It is the theories of physics that tell us that the states produced by decoherence are indistinguishable from those produced by a reduction. There is therefore a natural definition of when “something happens”. A measurement takes place when the state produced by decoherence is indistinguishable from a reduction according to the laws of physics [15]. No invocation of an external observer is needed. Measurements (more generally events) will be plentiful and happening all the time around the universe as quantum systems interact with the environment irrespective of whether or not an experimenter or measuring device is present. The resulting quantum theory can therefore be formulated on purely quantum terms, without invoking a classical world. It also naturally leads to a new ontology consisting of quantum systems, states and events, all objectively defined, in terms of which to build the world. One could ask: Were systems, states and events not already present in the Copenhagen interpretation? Could we not have used them already to build the world? Not entirely, since the definition of event used there required the existence of a classical external world to begin with. It therefore cannot be logically used to base the world on.

In this short review, we would like to outline some results supporting the above point of view. In the next section, we discuss how to use real clocks to describe physical systems where no external time is available. We will show that the evolution of the states presents a fundamental loss of coherence. Notice that we are not modifying quantum mechanics, just pointing out that we cannot entirely access the underlying usual unitary theory when we describe it in terms of real clocks (and measuring rods for space if one is studying quantum field theories). In the following section, we discuss how fundamental limitations of measurement prevent us from distinguishing a state produced by a reduction and a state produced by decoherence. Obviously, given the complexities of the decoherence process, we cannot show in general that this is the case. We will present a modification of a model of decoherence presented by Zurek [16] to analyze this type of situation to exhibit the point we are making. The next section discusses some philosophical implications of having a realist interpretation of quantum mechanics like the one proposed. We end with a summary.

## 2. Quantum Mechanics without an External Time

When one considers a system without external time, like when one studies cosmology, or model systems like a set of particles with fixed angular and linear momentum assuming no knowledge of external clocks (see [17] for references), one finds that the Hamiltonian does not generate evolution, but becomes a constraint that can be written generically as H=0. One is left with what is called a “frozen formalism” (see [18,19] and the references therein). The values of the canonical coordinates at a given time q(t),p(t) are not observable, since one does not have access to *t*. Physical quantities have to have vanishing Poisson brackets with the constraint; they are what is known as “Dirac observables”, and the canonical coordinates are not. The resulting picture is very different from usual physics, and it is difficult to extract physical predictions from it since the observables are all constants of the motion, as they have vanishing Poisson brackets with the Hamiltonian. People have proposed several possible solutions to deal with the situation, although no general consensus on a solution exists. We will not summarize all proposals here, in part because we will not need most of them and for reasons of space. We will focus on two proposals that, when combined, we claim provide a satisfactory solution to how to treat systems without external time when combined with each other. For other approaches, the review by Kuchař is very complete [19].

The first proposal we call “evolving Dirac observables”. It has appeared in various guises over the years, but it has been emphasized by Rovelli [20]. The idea is to consider Dirac observables that depend on a parameter O(t). These are true Dirac observables, they have vanishing Poisson brackets with the constraint, but their value is not well defined till one specifies the value of a parameter. Notice that *t* is just a parameter; it does not have to have any connection with “time”. The definition requires that when the parameter takes the value of one of the canonical variables, the Dirac observable takes the value of another canonical variable, for example, $Q\left(t=q_{1}\right)=q_{2}$. This in part justifies why it is a Dirac observable. Neither $q_{1}$ nor $q_{2}$ can be observed since we do not have an external time, but the value $q_{2}$ takes when $q_{1}$ takes a given value is a relation that can be defined without referring to an external time, i.e., it is invariant information. As an example, let us consider the relativistic particle in one dimension. We parameterize it, including the energy as one of the canonical variables, $p_{0}$. One then has a constraint $\phi =p_{0}^{2}-p^{2}-m^{2}$. One can easily construct two independent Dirac observables: *p* and $X\equiv q-p\;q^{0}/\sqrt{p^{2}+m^{2}}$ and verify that they have vanishing Poisson brackets with the constraint. An evolving constant of the motion could be,

(1) $$Q\left(t,q^{a},p_{a}\right)=X+\frac{p}{\sqrt{p^{2}+m^{2}}}t,$$

and one would have that when the parameter takes the value $q^{0}$, the evolving constant $Q\left(t=q^{0},q^{a},p_{a}\right)=q$ takes the value of one of the canonical variables. Therefore, one now has an evolution for the system, the one in terms of the parameter *t*. However, problems arise when one tries to quantize things. There, variables like $q_{1}$ become quantum operators, but the parameter remains unquantized. How does one therefore make sense of $t=q_{1}$ at the quantum level when the left member is a classical quantity and the right a quantum operator (particularly when the quantum operator is not a Dirac observable and therefore not defined on the physical space of states of the theory)?

The second approach was proposed by Page and Wootters [21]. They advocate quantizing systems without time by promoting all canonical variables to quantum operators. Then, one chooses one as a “clock” and asks relational questions between the other canonical variables and the clock. Conditional probabilities are well defined quantum mechanically. Therefore, without invoking a classical external clock, one chooses a physical variable as a clock, and to study the evolution of probabilities, one asks relational questions: what is the expectation value of variable $q_{2}$ when variable $q_{1}$ (which we chose as clock) takes the value 3:30 p.m.? Again, because relational information does not require the use of external clocks, it has an invariant character, and one can ask physical questions about it. However, trouble arises when one actually tries to compute the conditional probabilities. Quantum probabilities require computing expectation values with quantum states. In these theories, since we argued that the Hamiltonian is a constraint H=0, at a quantum level, one must have $\hat{H}|\Psi \rangle =0$; only states that are annihilated by the constraint are permissible. However, such a space of states is not invariant under multiplication by one of the canonical, variables, i.e., $\hat{H}q_{1}|\Psi \rangle \neq 0$. Therefore, one cannot compute the expectation values required to compute the conditional probabilities. One can try to force a calculation pretending that one remains in the space, but then one gets incorrect results. Studies of model systems of a few particles have shown that one does not get the right results for the propagators, for example [19].

Our proposal [13] is to combine the two approaches we have just outlined: one computes conditional probabilities of evolving constants of the motion. Therefore, one chooses an evolving constant of the motion that will be the “clock”, T(t), and then, one chooses a variable one wishes to study O(t) and computes,

(2) $$P\left(O\in \left[O_{0}-\Delta_{1},O_{0}+\Delta_{1}\right]|T\in \left[T_{0}-\Delta_{2},T_{0}+\Delta_{2}\right]\right)=\lim_{\tau \to \infty }\frac{\int_{-\tau }^{\tau }dt\mathrm{Tr}\left(P_{O_{0}}^{\Delta_{1}}\left(t\right)P_{T_{0}}^{\Delta_{2}}\left(t\right)\rho P_{T_{0}}^{\Delta_{2}}\left(t\right)\right)}{\int_{-\tau }^{\tau }dt\mathrm{Tr}\left(P_{T_{0}}^{\Delta_{2}}\left(t\right)\rho \right)},$$

where we are computing the conditional probability that the variable *O* takes a value within a range of width $2\Delta_{1}$ around the value $O_{0}$ when the clock variable takes a value within a range of width $2\Delta_{2}$ around the value $T_{0}$ (we are assuming the variables to have continuous spectra, hence the need to ask about ranges of values) on a quantum state described by the density matrix ρ. The quantity $P_{O_{0}}^{\Delta_{1}}$ is the projector on the eigenspace associated with the eigenvalue $O_{0}$ of the operator $\hat{O}$ and similarly for $P_{T_{0}}^{\Delta_{2}}$. Notice that the expression does not require assigning a value to the classical parameter *t*, since it is integrated over all possible values.

We have shown [13] using a model system of two free particles where we use one of them as the “clock” that this expression, provided one makes judicious assumptions about the clock, indeed reproduces to leading order the correct usual propagator, not having the problems of the Page and Wootters’ proposal.

The above expression in terms of conditional probabilities may look unfamiliar. It is better to rewrite it in terms of an effective density matrix. Then, it looks exactly like the ordinary definition of probability in quantum mechanics,

(3) $$P\left(O_{0}|T_{0}\right)=\frac{\mathrm{Tr}\left(P_{O_{0}}^{\Delta_{1}}\left(0\right)\rho_{\mathrm{eff}}\left(T_{0}\right)\right)}{\mathrm{Tr}\left(\rho_{\mathrm{eff}}\left(T_{0}\right)\right)},$$

where on the left-hand side, we shortened the notation omitting mention of the intervals, but they are still there. The effective density matrix is defined as,

(4) $$\rho_{\mathrm{eff}}\left(T\right)=\int_{-\infty }^{\infty }dtU_{s}\left(t\right)\rho_{s}U_{s}^{†}\left(t\right)P_{t}\left(T\right),$$

where we have assumed that the density matrix of the total system is a direct product of that of the subsystem we use as clock $\rho_{\mathrm{cl}}$ and that of the subsystem under study $\rho_{s}$, and a similar assumption holds for their evolution operators *U*. The probability,

(5) $$P_{t}\left(T\right)=\frac{\mathrm{Tr}\left(P_{T_{0}}^{\Delta_{2}}\left(0\right)U_{\mathrm{cl}}\left(t\right)\rho_{\mathrm{cl}}U_{\mathrm{cl}}^{†}\left(t\right)\right)}{\int_{-\infty }^{\infty }dt\mathrm{Tr}\left(P_{T_{0}}^{\Delta_{2}}\left(t\right)\rho_{\mathrm{cl}}\right)},$$

is an unobservable quantity since it represents the probability that the variable $\hat{T}$ take a given value when the unobservable parameter is *t*.

The introduction of the effective density matrix clearly illustrates what happens when one describes ordinary quantum mechanics in terms of a clock variable that is a real observable, not a classical parameter. Examining Equation (4), we see in the right-hand side the ordinary density matrix evolving unitarily as a function of the unobservable parameter *t*. If the probability $P_{t}\left(T\right)$ were a Dirac delta, then the effective density matrix would also evolve unitarily. That would mean that the real clock variable is tracking the unobservable parameter *t* perfectly. However, no physical variable can do that, so there will always be a dispersion, and the probability $P_{t}\left(T\right)$ will have non-vanishing support over a range of *T*. What this is telling us is that the effective density matrix for the system at a time *T* will correspond to a superposition of density matrices at different values of the unobservable parameter *t*. The resulting evolution is therefore non-unitary. We see clearly the origin of the non-unitarity: the real clock variable cannot keep track of the unitary evolution of quantum mechanics.

In fact, if we assume that the clock variable tracks the unobservable parameter almost perfectly by writing:

(6) $$P_{t}\left(T\right)=\delta \left(t-T\right)+b\left(T\right)\delta^{\prime \prime }\left(t-T\right)+\ldots ,$$

(a term proportional to $\delta {\left(t-T\right)}^{\prime }$ only adds an unobservable shift), one can show that the evolution implied by (4) is generated by a modified Schrödinger equation,

(7) $$-i\hbar \frac{\partial \rho }{\partial T}=\left[\hat{H},\rho \right]+\sigma \left(T\right)\left[\hat{H},\left[\hat{H},\rho \right]\right]+\ldots ,$$

where σ(T)=db(T)/dT is the rate of spread of the probability $P_{t}\left(T\right)$ and $\rho =\rho_{\mathrm{eff}}\left(T\right)$.

Therefore, we clearly see that when describing quantum mechanics in terms of a real clock variable associated with a quantum observable rather than with a classical parameter, the system loses unitarity, and it is progressively worse the longer one waits.

The existence of the effect we are discussing is not controversial. In fact, one can make it as large as one wishes simply choosing a bad clock. Bonifacio et al. [22,23,24] have reinterpreted certain experiments with Rabi oscillations as being described with an inaccurate clock, and indeed, experimentally, one sees the loss of coherence described above. More recently, it has been demonstrated with entangled photons, as well [25].

However, the question still remains: can this effect be made arbitrarily small by a choice of the clock variable? If one takes into account gravity, the answer is negative. Using non-gravitational quantum physics, Salecker and Wigner [10,11] examined the question of how accurate a clock can be. The answer is that the uncertainty in the measurement of time is proportional to the square root of the length of time one desires to measure and inversely proportional to the square root of the mass of the clock. Therefore, to make a clock more accurate, one needs to make it more massive. However, if one takes gravity into account, there clearly is a limitation as to how massive a clock can be: at some point, it turns into a black hole. Several phenomenological models of this were proposed by various authors, and they all agree that the ultimate accuracy of a clock goes as some fractional power of the time to be measured times a fractional power of Planck’s time [26,27,28,29,30]. Different arguments lead to slightly different powers, but the result is always that the longer one wishes to measure time, the more inaccurate the clocks become. For instance, in the phenomenological model of Ng and Van Dam [26,27,28,29,30], one has that $\delta T∼T^{1/3}T_{\mathrm{Planck}}^{2/3}$. Substituting that in the modified Schrödinger equation, its solution can be found in closed form, in an energy eigenbasis,

(8) $$\rho {\left(T\right)}_{nm}=\rho_{nm}\left(0\right)\exp\left(-i\omega_{nm}T\right)\exp\left(-\omega_{nm}^{2}T_{\mathrm{Planck}}^{4/3}T^{2/3}\right),$$

where $\omega_{nm}$ is the Bohr frequency between the two energy eigenstates *n* and *m*. We see that the off-diagonal terms of the density matrix die off exponentially. Pure states evolve into mixed states.

## 3. Completing Decoherence: The Montevideo Interpretation

### 3.1. Decoherence with Clocks Based on Physical Variables

In this section, we would like to analyze how the use of a physical clock in the description of quantum mechanics we introduced in the last section, combined with other limitations in measurement, will help address the objections to environmental decoherence as a solution to the measurement problem. We start by illustrating the idea of decoherence (and the objections) using a well-known model of environmental decoherence due to Zurek [16], possibly one of the simplest models one can consider that still captures the complexities involved.

#### 3.1.1. Zurek’s Model

It consists of a spin one half system representing the microscopic system plus the measuring device, with a two-dimensional Hilbert space {|+〉,|−〉}. It interacts with an “environment” given by a bath of many similar two-state “atoms”, each with a two-dimensional Hilbert space ${\{|+\rangle }_{k}{,|-\rangle }_{k}\}$. If there is no interaction with the environment, the two spin states may be taken to have the same energy; we choose it to be zero, and all the atoms also are chosen with zero energy. The interaction Hamiltonian is given by:

(9) $$H_{\mathrm{int}}=\hbar \sum_{k}\left(g_{k}\sigma_{z}⊗\sigma_{z}^{k}{⊗}_{j\neq k}I_{j}\right).$$

$\sigma_{z}$ is a Pauli matrix acting on the state of the system. It has eigenvalues +1 for the spin eigenvector |+〉 and −1 for |−〉. The operators $\sigma_{z}^{k}$ are similar, but acting on the state of the *k*-th atom. $I_{j}$ denotes the identity matrix acting on atom *j*, and $g_{k}$ is the coupling constant. It has dimensions of frequency and characterizes the coupling energy of one of the spins *k* with the system. The model can be thought physically as providing a representation of a photon propagating in a polarization analyzer.

Through the interaction, the initial state, which we can take as,

(10) $$|\Psi \left(0\right)\rangle =\left(a|+\rangle +b|-\rangle \right)\prod_{k=1}^{N}⊗\left[\alpha_{k}{|+\rangle }_{k}+\beta_{k}{|-\rangle }_{k}\right],$$

with $a,b,\alpha_{k}$ and $\beta_{k}$ complex constants, evolved using the Schrödinger equation, becomes,

(11) $$\begin{array}{ccc} |\Psi (t)\rangle & = & a|+\rangle \prod_{k=1}^{N}⊗\left[\alpha_{k}\exp\left(ig_{k}t\right){|+\rangle }_{k}+\beta_{k}\exp\left(-ig_{k}t\right){|-\rangle }_{k}\right]\\ & & +b|-\rangle \prod_{k=1}^{N}⊗\left[\alpha_{k}\exp\left(-ig_{k}t\right){|+\rangle }_{k}+\beta_{k}\exp\left(ig_{k}t\right){|-\rangle }_{k}\right]. \end{array}$$

From it, one can construct a density matrix for the system plus environment, and tracing out the environmental degrees of freedom, one gets a reduced density matrix for the system,

(12) $$\rho_{c}\left(t\right)={\left|a\right|}^{2}{\left|+\rangle \langle +|+|b\right|}^{2}|-\rangle \langle -|+z\left(t\right)ab^{*}|+\rangle \langle -|+z^{*}\left(t\right)a^{*}b|-\rangle \langle +|,$$

where:

(13) $$z\left(t\right)=\prod_{k=1}^{N}\left[\cos\left(2g_{k}t\right)+i\left(|\alpha_{k}{|}^{2}-{\left|\beta_{k}\right|}^{2}\right)\sin\left(2g_{k}t\right)\right].$$

The complex valued function of time z(t) determines the values of the off-diagonal elements. If it vanishes, the reduced density matrix could be considered a “proper mixture” representing several outcomes with their corresponding probabilities.

We claim that with the modified evolution we discussed in the previous section, the usual objections to decoherence do not apply. Recall which are the usual objections:

1. The quantum coherence is still there. Although a quantum system interacting with an environment with many degrees of freedom will very likely give the appearance that the initial quantum coherence of the system is lost (the density matrix of the measurement device is almost diagonal), the information about the original superposition could be recovered for instance carrying out a measurement that includes the environment. The fact that such measurements are hard to carry out in practice does not prevent the issue from existing as a conceptual problem.
2. The “and/or problem”: Since the density matrix has been obtained by tracing over the environment, it represents an improper, not proper, mixture: looking at Equation (12), there is no way to select (even in some conceptual sense) one of the components of the density matrix versus the others.

Let us discuss now the problem of revivals. In the model, the function z(t) does not die off asymptotically, but is multi-periodic; after a very long time, the off-diagonal terms become large. Whatever definiteness of the values of the preferred quantity we had won by the end of the measurement interaction turns out in the very long run to have been but a temporary victory. This is called the problem of revivals (or “recurrence of coherence”, or “recoherence”). This illustrates that the quantum coherence persists, it was just transferred to the environment and could be measured using global observables.

#### 3.1.2. A More Realistic Model and Real Clocks

To analyze the effects of limitations of measurement and the use of real clocks in detail, we will need to consider a more realistic model of spinning particles [31]; the previous model is too simple to capture the effect of the use of real clocks. Although this model is “almost realistic”, it has the property that the system, environment and measurement apparatus are all under control, as one would need to measure a global observable, for instance. It consists of a spin *S* in a cavity with a magnetic field pointing in the *z* direction. A stream of *N* “environmental” spins flows sideways into the cavity and eventually exits it, and the interactions last a finite time determined by the time spent in the cavity. The flow of particles that represents the environment is sufficiently diluted such that we can ignore interactions among themselves.

The interaction Hamiltonian for the *k*-th spin of the environment is,

(14) $${\hat{H}}_{k}={\hat{H}}_{k}^{B}+{\hat{H}}_{k}^{\mathrm{int}},$$

with,

(15) $${\hat{H}}_{k}^{B}=\gamma_{1}B{\hat{S}}_{z}⊗{\hat{I}}_{k}+\gamma_{2}B\hat{I}⊗S_{z}^{k},$$

and:

(16) $${\hat{H}}_{k}^{\mathrm{int}}=f_{k}\left({\hat{S}}_{x}{\hat{S}}_{x}^{k}+{\hat{S}}_{y}{\hat{S}}_{y}^{k}+{\hat{S}}_{z}{\hat{S}}_{z}^{k}\right),$$

where $f_{k}$ are the coupling constants between the spin and each of the particles of the environment, $\gamma_{1}$ and $\gamma_{2}$ are the magnetic moments of the central and environment spins, respectively, and the $\hat{S}$ are spin operators.

For the complete system, one can define an observable considered by d’Espagnat [3]. It has the property that its expectation value is different depending on if the state has suffered a quantum collapse or not. It definition is,

(17) $$\hat{M}\equiv {\hat{S}}_{x}⊗\prod_{k}^{N}{\hat{S}}_{x}^{k}.$$

One has that ${\langle \hat{M}\rangle }_{\mathrm{collapse}}=0$, whereas,

(18) $$\langle \psi |M|\psi \rangle =ab^{*}\prod_{k}^{N}\left[\alpha_{k}\beta_{k}^{*}+\alpha_{k}^{*}\beta_{k}\right]e^{-2i\Omega_{k}\tau }+a^{*}b\prod_{k}^{N}\left[\alpha_{k}\beta_{k}^{*}+\alpha_{k}^{*}\beta_{k}\right]e^{2i\Omega_{k}\tau }\neq 0,$$

with $\Omega_{k}\equiv \sqrt{4f_{k}^{2}+B^{2}{\left(\gamma_{1}-\gamma_{2}\right)}^{2}}$ and τ is the time of flight of the environmental spins through the chamber. One can therefore determine experimentally if a collapse or not took place measuring this observable.

However, if one considers the corrections to the evolution resulting from the use of physical variables as clocks as we discussed in the previous section, one has that,

(19) $$\begin{array}{c} \langle \hat{M}\rangle \;=\;ab^{*}e^{-i2N\Omega T}e^{-4NB^{2}{\left(\gamma_{1}-\gamma_{2}\right)}^{2}\theta }\prod_{k}^{N}\left[\alpha_{k}\beta_{k}^{*}e^{-16B^{2}\gamma_{1}\gamma_{2}\theta }+\alpha_{k}^{*}\beta_{k}\right]\\ \;\;\;\;\;\;\;\;\;\;\;\;\;\;\;\;+ba^{*}e^{i2N\Omega T}e^{-4NB^{2}{\left(\gamma_{1}-\gamma_{2}\right)}^{2}\theta }\prod_{k}^{N}\left[\alpha_{k}\beta_{k}^{*}+\alpha_{k}^{*}\beta_{k}e^{-16B^{2}\gamma_{1}\gamma_{2}\theta }\right], \end{array}$$

where $\Omega \equiv B(\gamma_{1}-\gamma_{2})$, $\theta \equiv \frac{3}{2}T_{P}^{4/3}\tau^{2/3}$, τ is the time of flight of the environment spins within the chamber and *T* is the length of the experiment.

There exists a series of conditions for the experiment to be feasible that imply certain inequalities,

(20) $$\begin{array}{c} \left(a\right)\;1<f\tau =\frac{\mu \gamma_{1}\gamma_{2}}{\hbar }\frac{\tau }{d^{3}}, \end{array}$$

(21) $$\begin{array}{c} \left(b\right)\;\Delta x∼\sqrt{\frac{\hbar T}{m}}, \end{array}$$

(22) $$\begin{array}{c} \left(c\right)\;f\ll |B\left(\gamma_{1}-\gamma_{2}\right)|, \end{array}$$

(23) $$\begin{array}{c} \left(d\right)\;\langle \hat{M}\rangle ∼\exp\left(-6NB^{2}{\left(\gamma_{1}-\gamma_{2}\right)}^{2}T_{\mathrm{Planck}}^{4/3}\tau^{2/3}\right), \end{array}$$

with *f* the interaction energy between spins, which was assumed constant through the cell, μ the permeability of the vacuum, *d* the impact parameter of the spins of the environment, *m* their mass and Δx the spatial extent of the environment particles.

Condition (*a*) makes the coupling of the spins strong enough for decoherence to occur; (*b*) is to prevent the particles of the environment from dispersing too much and therefore making us unable to find them within the detectors at the end of the experiment; (*c*) is the condition for decoherence to be in the *z* basis, as was mentioned; (*d*) is an estimation of the the expectation value of the observable when the effect of the real clock is taken into account. For details of the derivation of these conditions, see our previous paper [32].

Therefore, the expectation value is exponentially damped, and it becomes more and more difficult to distinguish it from the vanishing value one has in a collapse situation. A similar analysis allows one to show that revivals are also prevented by the modified evolution. When the multi-periodic functions in the coherences tend to take again the original value after a Poincaré time of recurrence, the exponential decay for sufficiently large systems completely hides the revival under the noise amplitude.

Thus, the difficulties found in testing macroscopic superpositions in a measurement process are enhanced by the corrections resulting from the use of physical clocks.

### 3.2. Why the Solution Is Not FAPP

Although temporal decoherence involves exponentials and the troublesome terms of decoherence become exponentially small, how does this observation help to solve the problem of outcomes? In what follows, we will provide a criterion for the occurrence of events based on the notion of undecidability.

When one takes into account the way that time enters in generally covariant systems including the quantum fluctuations of the clock, the evolution of the total system (system plus measurement apparatus plus environment) becomes indistinguishable from the collapse. This is also true for revivals and the observation of the coherences of the reduced density matrix of the system plus the measuring device. We call such a situation “undecidability”. We are going to show that undecidability is not only for all practical purposes (FAPP), but fundamental.

From the previous discussion, one can gather that as one considers environments with a larger number of degrees of freedom and as longer time measurements are considered, distinguishing between collapse and unitary evolution becomes harder. However, is this enough to be a fundamental claim?

Starting from (19) and using the approximations (20)–(23), one can show [32] that,

(24) $$\langle \hat{M}\rangle ∼\exp\left(-6NB^{2}{\left(\gamma_{1}-\gamma_{2}\right)}^{2}T_{\mathrm{Planck}}^{4/3}\tau^{2/3}\right)\equiv e^{-K}.$$

with

(25) $$K\gg \frac{N^{5}T_{\mathrm{Planck}}^{4/3}\hbar^{20/3}}{m^{4}{\left(\gamma_{1}\gamma_{2}\right)}^{8/3}\mu^{8/3}}.$$

Is it possible to build a very large ensemble allowing one to distinguish this value from zero?

Brukner and Kofler [33] have recently proposed that from a very general quantum mechanical analysis together with bounds from special and general relativity, there is a fundamental uncertainty in the measurements of angles even if one uses a measuring device of the size of the observable Universe.

(26) $$\Delta \theta ≳\frac{l_{P}}{R},$$

where $l_{P}\equiv \sqrt{\hbar G/c^{3}}\approx 10^{-35}m$. If we take the radius of the observable universe as a characteristic length, $R\approx 10^{27}m$, we reach a fundamental bound on the measurement of the angle,

(27) $$\Delta \theta \geq 10^{-62}.$$

To distinguish 〈M〉 from zero, one needs to take into account that the observable will have an error that depends on Δθ (since for instance ${\hat{S}}_{x}$ and ${\hat{S}}_{y}$ will get mixed). If the error is larger than 〈M〉, there is no way of distinguishing collapse from a unitary evolution *for fundamental, not practical reasons*. Therefore, the solution is not FAPP.

The expectation value of the observable is [32],

(28) $$\langle {\hat{M}}^{\Delta \theta }\rangle ≳e^{-K}\pm {\left(\Delta \theta \right)}^{2N}+\langle E\left(\Delta \theta \right)\rangle .$$

with:

(29) $$K\gg \frac{N^{5}T_{\mathrm{Planck}}^{4/3}\hbar^{20/3}}{m^{4}{\left(\gamma_{1}\gamma_{2}\right)}^{8/3}\mu^{8/3}}.$$

Therefore, for,

(30) $$N>{\left(\frac{2\ln\left(\frac{R}{\ell_{P}}\right){\left(m{\left(\gamma_{1}\gamma_{2}\right)}^{4}\right)}^{2/3}\mu^{8/3}}{T^{4/3}\hbar^{20/3}}\right)}^{1/4}m{\left(\gamma_{1}\gamma_{2}\right)}^{2}∼10^{7},$$

it becomes undecidable whether collapse has occurred or not. That means that no measurement of any quantity, even in principle, can ascertain whether the evolution equation failed to hold. Notice that the above discussion was restricted to a given experiment. Our present knowledge of quantum gravity and the complexities of the decoherence process in general does not allow us to prove undecidability for an arbitrary experimental setup. Even models slightly more elaborate than the one presented here can be quite challenging to analyze. A different model, involving the interaction of a spin with bosons, has also been analyzed with similar results [34].

This model exhibits the difficulties of trying to obtain generic results concerning decoherence. Notice that expression (30) depends on the magnetic moments of the spins $\gamma_{1,2}$. If they were very large, decoherence would not take place. One would be in the presence of a macroscopic system exhibiting quantum behavior. One does not expect such systems to exist, at least in the terms described in the model, but the model does not rule them out.

### 3.3. The Problem of Outcomes, Also Known as the Issue of Macro-Objectification

The problem of macro-objectification of properties may be described according to Ghirardi as follows: how, when and under what conditions do definite macroscopic properties emerge (in accordance with our daily experience) for systems that, when all is said and done, we have no good reasons for thinking they are fundamentally different from the micro-systems from which they are composed?

We think that undecidability provides an answer to this problem. We will claim that events occur when a system suffers an interaction with an environment such that one cannot distinguish by any physical means if a unitary evolution or a reduction of the total system, including the environment, took place. This provides a criterion for the production of events, as we had anticipated. In addition, we postulate (we call this the ontological postulate in [15]) that when an event occurs, the system chooses randomly (constrained by their respective probability values) one of the various possible outcomes. Having an objective criterion for the production of events based on undecidability answers the objections raised by [7] since the observer and the “super observer” now have consistent descriptions.

Philosopher Jeremy Butterfield, who has written an assessment of the Montevideo interpretation [35], has observed that up to now, we have only provided precise examples of undecidability for spinning particles. In that sense, he considers that the fundamental loss of coherence due to the use of quantum clocks and to the quantum gravitational effects should be used in the context of a many worlds interpretation because it helps to answer some of the long-held obstructions to the combination of the decoherence program with the many worlds approach.

After a detailed analysis, we do not believe that conclusion is inescapable. Let us assume the worse case scenario: that there are no further quantum gravitational limitations for the measurements of other variables as the ones obtained for the spin by Kofler and Bruckner (even though there have been many proposals to alter uncertainty relations; see the references in [36]). However, given the fact that the distinction between a unitary evolution that includes quantum time measurements or a quantum reduction would require an exponentially growing number of individual measurements in order to have the required statistics for distinguishing a non-vanishing exponentially small mean value from zero, limitations referring to the existence of a finite number of physical resources in a finite observable Universe would be enough to ensure undecidability. Obviously, further investigations are needed, but in a sense, this is the fate of all studies involving decoherence; it is just not possible to develop general proofs given the complexities involved.

## 4. Some Philosophical Implications

If the fundamental nature of the world is quantum mechanical and we adopt an interpretation that provides an objective criterion for the occurrence of events, we are led to an ontology of objects and events. The interpretation here considered makes reference to primitive concepts like systems, states, events and the properties that characterize them. Although these concepts are not new and are usually considered in quantum mechanics, one can assign them a unambiguous meaning only if one has an interpretation of the theory. For example, events could not be used as the basis of a realistic ontology without a general criterion for the production of events that is independent of measurements and observers.

On the other hand, the concepts of state and system only acquire ontological value when the events also have acquired it, since they are both defined through the production of events. Based on this ontology, objects and events can be considered the building blocks of reality. Objects will be represented in the quantum formalism by systems in certain states and are characterized by their dispositions to produce events under certain conditions. In the new interpretation, events are the actual entities. Concrete reality accessible to our senses is constituted by events localized in space-time. As Whitehead [37] recognized: “the event is the ultimate unit of natural occurrence”. Events come with associated properties. Events and properties in quantum theory are represented by mathematical entities called projectors. Quantum mechanics provides probabilities for the occurrence of events and their properties. When an event happens, like in the case of the dot on the photographic plate in the double-slit experiment, typically many properties are actualized. For instance, the dot may be darker on one side than the other, or may have one of many possible shapes.

Take for instance the hydrogen atom. It is a quantum system composed by a proton and an electron. A particular hydrogen atom is a system in a given state; it is an example of what we call an object, and it has a precise disposition to produce events. Russell in The Analysis of Matter [38], asserts that “the enduring thing or object of common sense and the old physics must be interpreted as a world-line, a causally related sequence of events, and that it is events and not substances that we perceive”. He thus distinguishes events as basic particulars from objects as derived, constructed particulars. We disagree with this point of view because it ignores the role of the physical states. He adds: “Bits of matter are not among the bricks out of which the world is built. The bricks are events and bits of matter are portions of the structure to which we find it convenient to give separate attention”. This is not the picture provided by quantum mechanics. An independent notion of object is required: one can even have event-less objects in quantum mechanics. For instance, when not measured, the hydrogen atom is an object according to the definition above even though it is not producing an event. The resulting ontology is such that objects and events are independent concepts; they are not derived one from the other.

This is only a sketch of philosophical issues raised by the new interpretation. We have a more complete discussion in [39].

## 5. Summary

We have presented an easy to follow guide to the Montevideo interpretation. Readers interested in an axiomatic formulation should consult our previous paper [15]. All the bibliography can be found in [9].

To summarize, the use of real physical variables to measure time implies a modification to how one writes the equations of quantum mechanics. The resulting picture has a fundamental mechanism for loss of coherence. When environmental decoherence is supplemented with this mechanism and taking into account fundamental limitations in measurement, a picture emerges where there is an objective, observer independent notion for when an event takes place. The resulting interpretation of quantum mechanics, which we call the Montevideo interpretation, is formulated entirely in terms of quantum concepts, without the need to invoke a classical world. We have been able to complete this picture for a simple realistic model of decoherence involving spins. Studies of more elaborate models are needed to further corroborate the picture.
